# Adherence to national guidance on foods and drinks to limit or avoid in pregnancy in England: The PEAR Study

**DOI:** 10.1017/S1368980024000600

**Published:** 2024-03-04

**Authors:** Lucy Beasant, Jenny Ingram, Pauline M. Emmett, Janet E. Cade, Caroline M. Taylor

**Affiliations:** 1 Centre for Academic Child Health, Bristol Medical School, University of Bristol, UK; 2 Nutritional Epidemiology Group, School of Food Science and Nutrition, University of Leeds, Leeds, UK

**Keywords:** Diet, pregnancy, midwife, nutrition guidance, public health, PEAR Study

## Abstract

**Objective:**

The NHS England website provides guidance on foods/drinks to avoid or limit in pregnancy because of microbiological, toxicological or teratogenic hazards. The aims were to determine adherence and whether demographic characteristics were associated with adherence.

**Design:**

Cross-sectional study.

**Setting:**

Online survey of postpartum women resident in England during pregnancy.

**Participants:**

Recently postpartum women resident in England during their pregnancy (n=598; median age 33 (IQR 30-36) years) completed an online questionnaire (April to November 2022). Questions included those on consumption of 21 food/drink items that the NHS advises pregnant women to avoid/limit. The study is part of the Pregnancy, the Environment And nutRition (PEAR) Study. Summary statistics were used to determine proportions adhering to the guidance. Adjusted logistic regression was used to model the associations of adherence with demographic characteristics.

**Results:**

Adherence was generally high (>90% for eight of ten food/drink items to be avoided). However, among pre-pregnancy consumers, several items were not completely avoided: e.g. 81% (128/158) for game meat/gamebirds, 37% (176/478) for cured meats pre-pregnancy, and 17% (81/467) for soft cheeses. Greater educational attainment (e.g. caffeinated soft drinks OR 2.25 (95% CI 1.28, 3.94)), greater maternal age (e.g. oily fish 1.64 (1.05, 2.56)) and lower parity (e.g. caffeinated coffee 0.28 (0.11, 0.69)) were the most usual characteristics associated with adherence.

**Conclusion:**

Evidence of concerning levels of non-adherence for some food/drink items suggest a case for more education on some of the guidance, particularly for women with lower educational attainment, greater parity and greater maternal age. Further research on barriers to the implementation of the guidance is needed.

## Introduction

During pregnancy, the guidance given to women in England is to follow a healthy diet broadly similar to that advised for the general population^(1)^. However, there is additional guidance regarding a number of food items for which pregnant women are advised to either limit or avoid consumption altogether^(1–10)^ (Supplementary Table 1). This guidance is based on several factors. Exposure to toxic metals and pollutants such as mercury, lead, dioxins and polychlorinated biphenyls (e.g. fish, game meat/gamebirds) is associated with a risk of adverse developmental effects including neurodevelopmental disorders^(11–14)^. Microbiological hazards such as listeria, toxoplasmosis and salmonella (e.g. unpasteurised milk, soft cheese, cured meats) can lead to miscarriage, premature birth and stillbirth^(15, 16)^. Excess provision of vitamin A (e.g. in liver and liver products) can cause teratogenesis^(17)^. Some herbal teas, including fennel, ginger, chamomile and peppermint, can have pharmacological actions or interactions with drugs^(18)^. Adherence to guidance can reduce the likelihood of these serious outcomes.

The main summary of guidance on foods/drinks to avoid or limit in pregnancy is provided an NHS website page^(3)^ for England and is disseminated directly through midwives and other healthcare professionals^(19)^, as well as through leaflets, apps (e.g. Emma’s Diary, Baby Buddy), other websites(^20, 21)^, and by word-of-mouth from friends and relatives. Studies on nutrition guidance in pregnancy have generally focused on healthy eating guidance and diet quality^(22–24)^, or on a particular age group^(25)^ or food item (e.g. fish^(26)^), or avoidance in response to traditional beliefs^(27)^. The few studies on specific foods to avoid or limit mainly focussed only on listeria^(28, 29)^. However, a broader study in Australia showed that knowledge of foods to avoid was poor^(30)^ while a study in New Zealand found that 12% of pregnant women did not avoid any particular food item^(31)^. Similarly, only 53% of women in a study in Canada followed food avoidance recommendations overall, but there were no data reported on individual food items^(32)^.

To date, there has not been a study to evaluate adherence to NHS guidance on foods/drinks to avoid or limit by pregnant women in England or an examination of sources of information about the guidance. This information could provide an evidence base to inform the future development of the content of the guidance and its dissemination in order to maximise its usability and beneficial impact. The primary aim therefore was to determine adherence to the NHS guidance on foods to avoid or limit during pregnancy in England, including changes in consumption from pre-pregnancy. The secondary aims were to determine the sources of information used by pregnant women to inform themselves about which foods/drinks to avoid or limit, and which sources they trusted most, and to determine if any demographic characteristics were associated with adherence.

## Methods

The study is part of a larger mixed methods study on dietary exposure to toxic metals (The Pregnancy, the Environment And nutRition (PEAR) Study)^(33)^. Recently postpartum women (≤12 months) resident in England for ≥6 months of their pregnancy were recruited to complete a custom-designed online questionnaire hosted on Jisc Online Surveys^(34)^. Ethics approval was given by the University of Bristol Health Sciences Research Ethics Committee (reference 106742, 21 April 2021). The main purpose of the questionnaire was to collect data on consumption of food items that the NHS advised pregnant women to avoid because of dietary exposure to toxic metals (mercury and lead).

### Questionnaire

The initial version of the questionnaire was tested with postpartum women (n=9) in an adapted ‘Think Aloud’ exercise and modified according to their feedback^(35)^. Participants were emailed a link to access the electronic questionnaire and answered each question in the presence of a researcher (LB). ‘Think Aloud’ discussions were conducted remotely via video or telephone call and were recorded using an encrypted digital audio-recorder. Participants were asked to ‘Think Aloud’ as they accessed and filled in the questionnaire, vocalising their thoughts about the questions, covering, for example, any comprehension issues, the acceptability of available answers and technical problems including skip rules and the order of questions. Three ‘practice questions’ were provided at the beginning of the questionnaire to ensure the participant understood what the exercise involved. Questions and queries from the participant were addressed by the researcher, who made brief field notes during the exercise and remained silent other than to politely encourage the participant to ‘keep thinking aloud’ if they fell silent. When the participant had completed the questionnaire, the researcher used notes made during the exercise to probe any area where the participant seemed uncertain. Development of the questionnaire was iterative, with alterations being made in response to the comments of up to five participants at a time, until data saturation was reached and no new issues were reported.

The finalised questionnaire was open from April to September 2021. Participants were recruited primarily through publicity with paid advertising boosts on a study Facebook page linked to the study website with direct access to the questionnaire from the website^(33)^. Informed consent to participate was assured by completion of the questionnaire. With the exception of the screening questions to determine eligibility, no questions were compulsory to maximise the completion rate. Participants were able to re-access their partially completed questionnaire so that they did not have to complete it in one session. Questions included those in the following categories.

(1) *Screening questions* (consent, location during pregnancy, age of baby).
(2) *Demographics* (e.g. geographical location, ethnicity, age, highest educational qualification, household income, parity). Where comparable data were available, the values were compared with the most recent values for the population in England (or the UK) to gauge the representativeness of the participants^(36–39)^.
(3) *Consumption of foods and drinks (before and during pregnancy)*. The items included were those listed in the NHS website with guidance to avoid during pregnancy (game meat/gamebirds, soft cheese, unpasteurised milk, pate (meat and vegetarian), cured meats, liver/liver products, alcohol, shark/marlin/swordfish, standard multivitamins) and those to limit (total fish, oily fish, fresh and canned tuna, caffeinated drinks, herbal tea). Two items that had previously had guidance on restriction but for which guidance has changed were also included (peanuts and hens’ eggs). The questionnaire did not include items that involved guidance on preparation or cooking methods (unwashed fruits and vegetables, uncooked shellfish, sushi, cooked rare meat, goose/duck eggs) or liquorice root. Consumption of omega-3 supplements, although not on the main NHS list of items to avoid, was included because they can contain high levels of vitamin A if derived from fish oil^(4)^. We did not include a question on cooking smoked fish or sushi as this guidance was posted in response to a listeria outbreak in England linked to uncooked smoked fish after the survey had closed. For most dietary items participants were provided with six options for consumption of each during pregnancy: Ate or drank it more often during pregnancy than before/Ate or drank it or same during pregnancy than before/Ate or drank it less often during pregnancy than before/Ate or drank it before pregnancy but avoided it during pregnancy/Did not eat or drink it anyway/Don’t know or Can’t remember. For shark/marlin/swordfish, tinned tuna, fresh tuna and oily fish, participants were provided with the following six options for consumption during pregnancy: Never/More than once per month/1-2 times per month/Once per week/Several times per week/Don’t know or Can’t remember. For standard multivitamins and omega-3 supplements, the options for consumption during pregnancy were: Never/Less than once per month/1-2 times per month/About once a week/Several times a week/Once a day/Don’t know or can’t remember.
(4) *Sources of information about the guidance* (e.g. midwife or other healthcare professional, NHS website, other websites, leaflets, apps, friends and relatives). Participants were also asked to provide free text on which sources of information they trusted and which they felt less confident in. The questions in this section allowed for multiple answers to be given.

### Data analysis

Data were analysed with IBM SPSS Statistics version 26. Analyses were undertaken in two groups of participants: (1) all participants; (2) pre-pregnancy consumers only. (The all-participants group includes, for example, those who were vegetarian or vegan and did not eat fish even before pregnancy, so are not specifically following the guidance on this during pregnancy, but rather continuing with a dietary preference. The pre-pregnancy group consumers only group eliminates this group and this considers only those for whom the guidance is directly relevant.) To identify pre-pregnancy consumers only for each item, cases were filtered out by de-selecting cases: (1) if ‘Never’ or ‘Don’t know/Can’t remember’ was selected for the question about how much of the item they ate pre-pregnancy for game meat/gamebirds, fish, oily fish, tinned tuna, fresh tuna and shark/marlin/swordfish; or (2) if ‘Don’t eat/drink anyway’ or ‘Don’t know/Can’t remember’ was selected for cured meats, soft cheese, unpasteurised milk, alcohol, pate, liver/liver products, caffeinated drinks, herbal tea, hens’ eggs and peanuts.

The demographic characteristics of all participants were analysed with summary statistics and compared with national data where available.

The percent adhering to the guidance in all participants was calculated after exclusion of those responding ‘Don’t know/Can’t remember’, as well as in subgroups of pre-pregnancy consumers, using one-sample binomial success rate (Clopper-Pearson exact confidence intervals) to determine the proportions (%) and 95% confidence intervals. Categorisations of adherence (Yes/No) are shown in Supplementary Table 2.

The changes in the frequency of consumption of the specific food and drink items (before and during pregnancy) were also summarised for all participants and for pre-pregnancy consumers only.

The associations between changes in consumption frequencies and age (<30/≥30 years), parity (1/≥1), household income (<£30,000/≥£30,000, highest education attainment (Low (None/GCSE/Vocational level 1 and 2/AS or A level/Vocational level 3)/High (University degree (BSc, BA)/Professional qualification/Vocational levels 4 and 5/University higher degree (MA, MSc, PhD)), and following a special diet (Yes/No)) were determined (Chi-squared test).

Logistic regression was used to model the odds (95% confidence intervals) of adhering versus not adhering to guidance for each item adjusting for Education (None/GCSE/A levels/Vocational 1-3, Degree/Higher Degree/Vocational 4-5), Maternal age (18-25, >25-35, >35 years), Household income (≤£50,000, >£50,000), Region (North: North East/North West/Yorkshire and Humberside; Midlands: East Midlands/West Midlands; South: East/Greater London/South East/South West), Parity (1, >1), Special diet (No, Yes), Maternal age (18-25, >25-35, >35 years), Ethnicity (White, Other)). The regression analyses were done in all participants and in pre-pregnancy consumers only.

## Results

The questionnaire was accessed by 2751 respondents of whom 15 were screened out as ineligible (≥12 months postpartum and/or resident in England for ≤6 months of their pregnancy). The survey was completed by 598 participants (2034 accessed the initial information pages only; a further 20 did not progress beyond the eligibility screening pages; completion rate 85% for those that progressed beyond the eligibility screening pages). The demographics of the participants are shown in Table 1. The participants mean age was similar to the mean maternal age at birth in England and Wales in 2017^(38)^. All regions of England were represented and values for the regions in three categories (North, Midlands, South) were similar to national values^(37)^. However, the participants were more highly educated and had a higher household income than nationally, and were more likely to have ‘White’ rather than ‘Other’ ethnicity and have a parity of 1 rather than ≥1^(36, 37)^. Most had undertaken paid work during their pregnancy and all had home internet access. Twenty percent (122/598) followed a particular diet or diets (vegetarian no fish 6% (36/598), vegetarian with fish 2% (14/598), vegan 3% (16/598), low carb 3% (18/598), flexitarian 2% (9/598), gluten/wheat free 5% (28/598), low calorie 2% (11/598), other (including FODMAP, Paleo/Atkins, soy free, low sugar, other) 2% (12/598)).

In all participants, adherence was >90% for 8 of the 10 food/drinks to avoid with the exception of soft cheese (86%) and cured meats (71%). In pre-pregnancy consumers only, adherence was >90% for only 2 of the 10 items (liver/liver products and paté) (Table 2). For food/drinks with an advised limit, adherence was less prevalent in all participants, with only 5 of 9 items having adherence of >90%, but 4 of 9 items >90% in pre-pregnancy consumers (Table 2).

Changes in the frequency of consumption of food and drink items listed in the NHS website to avoid or limit during pregnancy compared with before pregnancy are shown in Tables 3 and 4. 37% (176/478) of consumers of cured meats pre-pregnancy did not then avoid cured meats in pregnancy and 17% (81/467) of consumers of soft cheeses pre-pregnancy did not avoid soft cheeses in pregnancy. 81% (128/158) of consumers of game meat/gamebirds pre-pregnancy did not avoid them during pregnancy.

For herbal teas (for which guidance is to limit to no more than four cups per day) there was an increase in consumption with 33% of all participants drinking more during pregnancy.

Changes in the frequencies of consumption of several food items to avoid from before pregnancy to during pregnancy were frequently associated with higher educational attainment and household income (Supplementary Table 3), but infrequently with parity and not with region of England. Associations with having a special diet were confined to food items containing meat, reflecting the relatively high proportion of self-reporting vegans and vegetarians (8%) (National Diet and Nutrition Survey (NDNS) value 2.3% in a representative UK population sample)^(40)^.

The most usual characteristic that predicted adherence for the 21 food/drink items in all participants was greater educational attainment for 4 items, 2 of which were caffeinated drinks (caffeinated soft drinks odds ratio (OR) 2.25 (95% confidence interval (CI) 1.28, 3.94), caffeinated tea OR 3.53 (95% CI 1.70, 7.40), oily fish OR 2.06 (95% CI 1.03, 4.12), hens’ eggs (OR 1.94 (95% CI 1.08, 3.47); Supplementary Table 4). Greater maternal age predicted adherence for 3 items (fish OR 1.51 (95% CI 1.02, 2.25), oily fish OR 1.64 (95% CI 1.05, 2.56), hens’ eggs OR 1.50 (95% CI 0.92, 2.42)) but non-adherence for 1 (paté OR 0.37 (95% CI 0.17, 0.83)). Increasing parity was associated with non-adherence for 4 items, 3 of which were caffeinated drinks (caffeinated soft drinks (OR 0.51 (95% CI 0.31, 0.84), caffeinated tea OR 0.47 (95% CI 0.24, 0.92), caffeinated coffee OR 0.28 (95% CI 0.11, 0.69), standard multivitamins OR 0.38 (95% CI 0.16, 0.88)). The most frequently predicted item was tea (by education, parity and ethnicity: OR 3.53 (95% CI 1.70, 7.40), OR 0.47 (95% CI 0.24, 0.92), OR 0.27 (95% CI 0.09, 0.81), respectively). The patterns were similar in participants who were consumers pre-pregnancy.

The main sources of information for women specifically in relation to fish were online (cited by 72%), verbal information (24%) and leaflets (16%). Apps were cited by 6% of participants, and magazines or books by 3%. Of those that accessed information online, the majority cited the NHS website (93%) with other sources including Mumsnet (8%), Tommy’s (7%), Facebook (4%) BBC website (1%) and The Pregnancy Book online (2%). The most popular app among users was Bounty (39%). Others included Pregnancy+ (31%), Emma’s Diary (27%), Oviva (20%) and Baby Buddy (12%). Of those that received verbal information, 57% cited a midwife at the general practitioners (GP), 25% a midwife at the hospital and 18% a midwife elsewhere. Other sources of information were relatives (15%), friends (15%), doctors (4%) and childbirth classes (10%). Leaflets were sourced from the community midwife (46%), midwife at the hospital (25%), midwife elsewhere (29%), with 0% from the GP surgery or hospital clinic. 159 participants added free text about their most trusted source of information: 65% (104/159) cited the NHS website and 18% (29/159) midwives. Sources that participants felt less confident in included the internet and social media (particularly US websites, forums and blogs), apps, magazines and word of mouth.

## Discussion

This is the first study to our knowledge to quantify adherence to guidance on foods to avoid or limit in pregnancy in a large number of recently postpartum women in England. We found that adherence to the key messages was generally good (>90% in the group of all participants for 8 of 10 food/drink items for which avoidance is recommended), but there were a few food or drink items for which there was a concerning level of non-adherence, particularly in participants who had consumed the items before pregnancy. These include herbal teas, game meat/gamebirds, cured meats and soft cheese. Adherence to advice to eat at least two portions of fish per week, of which one should be oily, was also poor^(41)^. In a similar study in New Zealand with 458 women, the prevalence of avoidance of alcohol was similar to that in the present study (8% and 9% in New Zealand and England, respectively) but in New Zealand a greater proportion (14%) did not avoid raw (unpasteurised) milk^(31)^, the corresponding value in the present study being 2%. However, like-for-like comparisons are made difficult by variations in the guidance in different countries (for example, New Zealand advises against pre-packaged and ready-made salads^(42)^, which is not specifically advised against in England).

Non-adherence to guidance on foods to avoid or limit in pregnancy can have serious consequences. Soft cheeses and cured meats can carry listeria: in 2019, for example, pregnancy-associated cases of listeria accounted for 18% of all cases and one-third of these cases resulted in stillbirth or miscarriage^(43)^. Herbal teas may contain components with pharmacological action as well as having the potential for herb–drug interactions^(18, 44)^. Lead exposure, which can occur from consumption of lead-shot birds or meat in pregnancy, is associated with adverse neurodevelopmental outcomes in the offspring^(11–14)^.

Information provided on the NHS website was a key source of information on foods to limit or avoid for these pregnant women in England with home internet access. They also reported that midwives were important in delivering information on these foods, particularly in primary care. Both these sources were highly trusted. Participants in this study required internet access but pregnant women with less internet connectivity may rely more on direct contact from healthcare workers. The importance of the delivery of messages by local healthcare workers was also suggested from a study in Australia where greater knowledge of foods to avoid was associated with more general practice visits for antenatal care and fewer tertiary visits^(30)^. Similarly, in New Zealand, women reported that dietary changes in pregnancy were mainly influenced by national guidance and health professionals^(31)^. The timing of delivery of information may also be critical as influences on dietary choices change during pregnancy^(45)^.

The drivers of dietary change in pregnancy particularly in relation to foods to avoid or limit have been little studied. Concern for the baby’s health and to satisfy cravings may be important: these were the main reasons for changes made by women to their diet during pregnancy in Canada, which included changes to align with recommendations for caffeine, alcohol, milk, fruit and food safety^(32)^ (the participants increased their intakes of milk products, fruit and sweet items and decreased or eliminated caffeine, alcohol and meat). However, their changes to meat and fish intakes were contrary to recommendations. Specifically for fish, intakes in pregnancy in Australia were influenced by risk aversion in a context of fish as part of healthy diet, cost, personal taste, and confidence in choosing and preparing fish^(26)^. More generally, food cravings, increased appetite and improved taste of the food were the drivers of increased intakes of milk/dairy products, vegetables, fruit and fruit juices, bread/cereal and chocolate in the diet of pregnant adolescents in the USA, while altered taste and nausea drove decreased intakes of other items ^(46)^.

Our results indicated that increasing parity and lower educational attainment were associated with non-adherence on foods to avoid or limit, suggesting that advice on guidance could be targeted towards these groups of women. Similarly, an international systematic review of adherence to nutritional guidance during pregnancy indicated that women with higher educational attainment, older age and non-smoking were more like to be adherent^(22)^. Conversely, there were few associations with income, special diet or ethnicity, suggesting that these are unimportant in targeting advice. However, participants with low income and those of diverse ethnicity were under-represented in the present study and this requires further investigation. Barriers to the delivery of health-related guidance to women preconceptually in the UK have been shown to include lack of healthcare resources, lack of staff training, and the policies and procedures of the provider organisation^(47)^, and there are likely to be similar barriers during pregnancy. Specifically for listeria, Canadian healthcare providers were identified as a valuable and trusted source of information but women noted that the providers had limited time in appointments to discuss food safety^(28)^. The women turned instead to books, the internet (including government websites) and social networks. In an additional qualitative study with midwives, we identified that midwives were often not confident about their ability to provide accurate advice on the guidance and their recall of information was often mistaken^(48)^. The midwives expressed a need for additional training and access to resources, together with sufficient time in appointments to discuss the guidance.

For items for which adherence was relatively poor, the guidance may need more clarity and/or improved dissemination, as has been noted previously specifically for listeria^(28)^. For example, an understanding of which cured meats to avoid requires a distinction to be made between cooked cured meats (such as corned beef and cooked ham) which do not need to be avoided, and uncooked cured meats (such as salami, chorizo and prosciutto ham) which do need to be avoided. With regard to soft cheese, the guidance includes a level of complexity that may make it difficult to understand: it advises against: (1) ‘*any other foods made from unpasteurised milk, such as soft ripened goats’ cheese’; (2)* ‘*pasteurised or unpasteurised mould-ripened soft cheeses with a white coating on the outside, such as Brie, Camembert and chèvre (unless cooked until steaming hot)’*; (3) ‘*pasteurised or unpasteurised soft blue cheeses, such as Danish blue, Gorgonzola and Roquefort (unless cooked until steaming hot)’*. For individuals eating game meat/game birds it may be difficult to know if the item has been lead-shot, although recently some supermarkets have stopped stocking lead-shot meat and birds^(49)^. Although game meat/gamebirds were eaten by relatively few participants, those that did so pre-pregnancy were likely to continue to eat them during pregnancy. For fish, the guidance requires identification of fish species, knowledge of what is an oily versus a white fish, and a tally of weekly consumption. Barriers to fish consumption in the study have been explored more fully in additional qualitative work, but include confusion over specific details of the guidance^(41)^. However, even having knowledge of the guidance may be insufficient to prevent consumption: in Ireland 82% of mothers knew that certain foods should be avoided but 55% consumed high-risk foods for listeria, which included soft cheeses, in pregnancy^(29)^. Labelling of supermarket and menu items such as game, cured meats, soft cheeses, multivitamins and omega-3 supplements to show whether they are ‘pregnancy-friendly’ could help women to make informed choices, analogous to the UK nutrition information labelling system^(50, 51)^.

In addition, some guidance may also be difficult to locate on the website, or not referred to directly. For example, although NHS guidance to avoid high-dose multivitamin supplements or any supplements with vitamin A in them in pregnancy^(3)^ is clearly shown on the main web page, fish liver oil supplements which also contain high levels of vitamin A are not mentioned. Instead the NHS guidance advising against taking them in pregnancy is on a separate web page from the main guidance on foods to avoid in pregnancy^(4)^. We found that 14% of women took omega-3 supplements, which are not mentioned specifically in the guidance. Most types of omega-3 supplements are safe in pregnancy (e.g. derived from fish oil, krill oil, algal oil or flax seed oil), but those obtained from fish liver oil should be avoided because of its vitamin A content.

We were able to include a relatively large population of recently postpartum women (our sample includes about 0.1% of the live births in England plus Wales in 2021^(52)^) and the data are the first to our knowledge to assess adherence to NHS guidance on foods to avoid or limit in England. There are several limitations to our study, however. Some of the questions in the questionnaire were designed primarily to collect data on food frequency rather than adherence to the guidance directly. The study related specifically to the guidance for England and is not generalisable to other countries where guidance may differ in content and presentation. Our participants were not representative of the population in England, although the demographic comparisons made were largely with the general adult population and not specifically pregnant women. In particular, the participants all had access to the internet at home and were more highly educated than the general population. Non-white participants were under-represented, so we were unable to assess whether the guidance is culturally appropriate for these women. It is possible that many pregnant women would have less access to guidance on diet during pregnancy than the participants. For game meat/gamebirds, we were not able to distinguish whether the items were lead-shot or not, but this may not have been known by the participants either. The questionnaire item on ‘soft cheese’ and ‘cured meats’ may not have allowed participants to distinguish between specific ‘safe’ and ‘not advised’ soft cheese or cured meats in their responses. Similarly, we have no knowledge of the vitamin A content of the standard multivitamins or source of the oil in the omega-3 supplements, nor of the exact number of cups of herbal tea. Some women may have avoided specific foods or drinks for reasons unrelated to the guidance (for example, pregnancy sickness). The pregnancies spanned a period of time when many restaurants, a frequent source of game meat/gamebirds in our participants, were closed due to COVID restrictions, which may have altered usual consumption patterns. This study indicates that there is a need for further in depth work on women’s food and drink choices in pregnancy.

## Conclusion

We have shown evidence of concerning levels of non-adherence to guidance on avoiding or limiting food/drink items in pregnancy in this study, particularly for cured meats, herbal teas, soft cheeses and game meat/gamebirds. Some of the guidance on foods/drinks to avoid or limit is complex, and there is a case for more prominent publicity and clarification for some of the guidance, particularly for women with lower educational attainment and greater parity. The NHS website is a key source of trusted information on diet for pregnant women but may need updating with regard to omega-3 and fish liver oil supplements. Previous work has identified that delivery of dietary information by midwives, at the most effective time, as a trusted source of information, needs to be supported by appropriate training and access to resources. Further research on barriers to the delivery of the guidance to and its implementation by pregnant women is needed.

## Supplementary Material

Supplementary Tables

## Figures and Tables

**Table 1:** Demographic characteristics of postpartum women who completed the online questionnaire

Characteristic	n	Value	National indicator^(36–39)^
Age (years)	548	Range 21-46, median 33 (IQR 30-36)	Mean maternal age at birth 30.5
Home location	598		
North East/North West/Yorkshire and Humberside		153 (26%)	28%
East Midlands/West Midlands		106 (18%)	20%
East/Greater London/South East/South West		339 (57%)	53%
Highest educational attainment	596		
None/GCSE/Vocational level 1 and 2/AS or A level/Vocational level 3		114 (19%)	50%
University degree (BSc, BA)/Professional qualification/Vocational levels 4 and 5/University higher degree (MA, MSc, PhD)		482 (81%)	50%
Household income	561		
<£30,000		89 (16%)	50%
≥£30,000		472 (84%)	50%
Parity	597		
1		432 (72%)	42%
>1		165 (28%)	58%
Ethnicity	593		
White		563 (95%)	80%
Other		30 (5%)	20%
Age of baby (months)	598		
0-5		371 (62%)	
6-12		227 (38%)	
Followed a special diet before pregnancy	598		
Yes		122 (20%)	
No		476 (80%)	
Paid work during pregnancy	598		
Yes		547 (92%)	
No		51 (9%)	
Smoking during pregnancy	596		
No		576 (97%)	
Yes		20 (3%)	
Home internet access	598		
Yes		598 (100%)	
No		0 (0%)	

Values are n (%).

Adapted from Beasant *et al*. ^(41)^.

**Table 2:** Adherence to guidance on foods to avoid or limit in pregnancy (% (95% CI))

	Adherence to the guidance during pregnancy			
	All participants^i^		Pre-pregnancy-consumers only^j^	
	Yes (n)/No (n)	% (95% CI)	Yes (n)/No (n)	% (95% CI)
**Foods/drinks to avoid**				
Cured meats^a^	421/176	71 (67, 74)	302/176	63 (58, 68)
Game meat^b^	543/52	91 (89, 93)	108/50	68 (61, 75)
Gamebirds^b^	569/26	96 (94, 98)	95/24	80 (72, 87)
Soft cheese^a^	515/81	86 (83, 89)	386/81	83 (79, 86)
Unpasteurised milk^a^	583/14	98 (96, 99)	82/14	85 (77, 92)
Shark/marlin/swordfish^b^	585/5	99 (98, 100)	40/5	89 (76, 96)
Alcohol^a^	543/54	91 (88, 93)	446/54	89 (86, 92)
Paté (meat/vegetarian)^a^	568/29	95 (93, 97)	315/29	92 (88, 94)
Liver/liver products^a^	576/16	97 (96, 98)	180/16	92 (87, 95)
Standard multivitamins^c^	450/28	94 (92, 96)	-	-
**Foods/drinks to limit**				
Caffeinated drinks^d^				
Soft drinks	497/101	83 (80, 85)	357/101	78 (74, 82)
Tea	550/47	92 (90, 94)	399/47	89 (86, 92)
Coffee	575/25	96 (94, 97)	367/25	94 (92, 96)
Energy drinks	592/2	100 (99, 100)	88/2	98 (95, 100)
Herbal tea^d^	308/287	52 (48, 56)	85/287	23 (21, 29)
Fish^e^	157/438	26 (23, 30)	151/347	30 (26, 34)
Oily fish^f^	118/478	20 (17, 23)	114/291	28 (24, 33)
Tinned tuna^g^	581/12	98 (97, 99)	407/11	97 (95, 99)
Fresh tuna^g^	587/0	100 (100, 100)	157/0	100 (100, 100)
**Foods/drinks for which advice was previously to limit or avoid**				
Hens’ eggs^h^	496/99	83 (80, 86)	451/99	82 (78, 85)
Peanuts^h^	545/46	92 (90, 94)	439/46	91 (87, 93)

a Yes=(Ate or drank before pregnancy but avoided during pregnancy/Don’t eat or drink anyway); No=(Ate or Drank more/Ate or Drank same amount/Ate or Drank less).

b Yes=(Never); No=(Less than once a month/About one to two times per month/About once per week/Several times per week).

c Yes=(Never); No=(Less than once a month/About one to two times per month/About once per week/Several times per week/Once a day).

d Yes=(Drank less/Drank before pregnancy but avoided during pregnancy/Don’t drink anyway); No=(Drank more/Drank same amount).

e Yes=(Twice a week/More than twice a week); No=(Never/Less than twice a week).

f Yes=(About once a week); No=(Never/Less than once a month/About one to two times a month/Several times a week).

g Yes=(Never/Less than once a month/About one to two times a month/About once a week); No=(Several times a week).

h Yes=(Don’t eat anyway/Ate same amount/Ate more); No=(Ate less/Ate before pregnancy but avoided during recent pregnancy).

i Participants responding ‘Don’t know/Can’t remember’ were excluded from analysis.

j Cases were filtered out for analysis of consumers only by de-selecting cases for foods/drinks for game meat/gamebirds, fish, oily fish, tinned tuna, fresh tuna and shark/marlin/swordfish if they responded ‘Never’ or ‘Don’t know/Can’t remember’ to a question about how much of the item they ate pre-pregnancy. For cured meats, soft cheese, unpasteurised milk, alcohol, pate, liver/liver products, caffeinated drinks, herbal tea, hens’ eggs and peanuts cases were de-selected if the option ‘Don’t eat/drink anyway’ during pregnancy was selected.

**Table 3:** Change in intake of foods and drinks with guidance on avoiding consumption from before to during pregnancy (maximum n=598)

	All participants					Pre-pregnancy-consumers only			
	n	Don’t eat/drink anyway	Ate/drank same or more often^d^	Ate/drank less often	Ate/drank before but avoided	n	Ate/drank same or more often^d^	Ate/drank less often	Ate/drank before but avoided
Soft cheese	596	129 (22%)	26 (4%)	55 (9%)	386 (65%)	467	26 (5%)	55 (12%)	386 (83%)
Unpasteurised milk	597	501 (84%)	7 (1%)	7 (1%)	82 (14%)	96	7 (7%)	7 (7%)	82 (85%)
Liver/liver products	592	396 (67%)	6 (1%)	10 (2%)	180 (30%)	196	6 (3%)	10 (5%)	180 (92%)
Paté (meat/vegetarian)	597	253 (42%)	10 (2%)	19 (3%)	315 (53%)	344	10 (3%)	19 (6%)	315 (92%)
Game meat/gamebirds	594	436 (73%)	83 (14%)	45 (8%)	30 (5%)	158	83 (53%)	45 (28%)	30 (19%)
Cured meats	597	119 (20%)	80 (13%)	96 (16%)	302 (51%)	478	80 (17%)	96 (20%)	302 (63%)
Alcohol	597	97 (16%)	1 (0%)	53 (9%)	446 (75%)	500	1 (0%)	53 (11%)	446 (89%)
Shark/marlin/swordfish^a, b^	590	-	-	-	-	45	-	-	40 (89%)
Standard multivitamins^c^	478	(450 (94%))	-	-	-	-	-	-	-

For full details of guidance on foods/drinks to avoid in pregnancy see NHS website pages ^(1–10)^.

Participants responding ‘Don’t know/Can’t remember’ were excluded from analyses.

a 52/598 (9%) of participants did not include fish in their diet because they were vegan or vegetarian with no fish.

b Frequency of consumption of shark/marlin/swordfish during pregnancy: Never 585 (99%); About one to two times per month/About once a week/Several times a week 0 (0%)/Less than once per month 5 (1%).

c Frequency of standard multivitamin consumption during pregnancy: Never 450 (94%); Less than once per month/About one to two times per week/Several times a week 10 (2%); Once a day 18 (4%).

d Data for response categories ‘Ate/drank same’ and ‘Ate/drank more often’ were merged because of low numbers (<5) in the latter category.

**Table 4:** Change in intake of foods and drinks with guidance on limiting consumption from before to during pregnancy (maximum n=598)

	All participants						Pre-pregnancy-consumers only				
	n	Don’t eat/drink anyway	Ate/drank more often^b^	Ate/drank same^b^	Ate/drank less often^b^	Ate/drank before but avoided^b^	n	Ate/drank more often^b^	Ate/drank same^b^	Ate/drank less often^b^	Ate/drank before but avoided^b^
Fish^a b^	592	88 (15%)	86 (15%)	261 (44%)	135 (23%)	22 (4%)	504	86 (17%)	261 (52%)	135 (27%)	22 (4%)
Caffeinated drinks											
Coffee	598	206 (34%)	0 (0%)	25 (4%)	170 (28%)	197 (33%)	392	0 (0%)	25 (6%)	170 (43%)	197 (50%)
Tea^e^	597	151 (25%)	47 (8%)		238 (40%)	161 (27%)	446	47 (11%)		238 (53%)	161 (36%)
Soft drinks	598	140 (23%)	23 (4%)	78 (13%)	228 (38%)	129 (22%)	458	23 (5%)	78 (17%)	228 (50%)	129 (28%)
Energy drinks^e^	594	504 (85%)	(0%)		21 (4%)	67 (11%)	90	(1%)		21 (23%)	67 (74%)
Herbal tea	595	223 (37%)	195 (33%)	92 (15%)	60 (10%)	25 (4%)	372	195 (52%)	92 (25%)	60 (16%)	25 (7%)
Hens’ eggs^c^	595	45 (8%)	100 (17%)	351 (59%)	80(13%)	19 (3%)	549	100 (18%)	351 (64%)	80 (15%)	19 (3%)
Peanuts^d^	591	106 (18%)	63 (11%)	376 (64%)	30 (5%)	16 (3%)	485	63 (13%)	376 (78%)	30 (6%)	16 (3%)

Participants responding ‘Don’t know/Can’t remember’ were excluded from analysis.

a 52/598 (9%) did not include fish in their diet because they were vegan or vegetarian with no fish.

b Oily fish: Never 232 (39%); Less than once per month/About one to two times per month 231 (39%); About once per week/Several times per week 133 (22%). Tinned tuna: Never 216 (36%); Less than once per month/About one to two times per month 270 (45%); About once per week/Several times per week 107 (18%). Fresh tuna: Never 537 (91%); Less than once per month/About one to two times per month 50 (9%); About once per week/Several times per week 0 (0%).

c Guidance changed in 2019 from ‘*avoid eating runny or raw hens’ eggs’* to ‘*avoid raw or partially cooked hens’ eggs unless British Lion eggs or produced under Laid in Britain scheme’*.

d Guidance changed in 2009 from ‘*avoid eating peanuts especially if there family history of allergy’* to ‘*safe to eat unless nut allergy’*.

e Data for response categories ‘Ate/drank same’ and ‘Ate/drank more often’ were merged because of low numbers (<5) in the latter category.

## Data Availability

Underlying data are subject to an embargo until the end of the study funding in 2025. The data will then be made available to bona fide researchers on application from data.bris.ac.uk/data.
