# Supplementary material for: Adherence to national guidance on foods and drinks to limit or avoid in pregnancy in England: The PEAR Study
Source: Public Health Nutr. Author manuscript; Available in PMC 2024 Apr 15. (PMC11010152; doi:10.1017/S1368980024000600)
Supplement: Supplementary Tables [file EMS194530-supplement-Supplementary_Tables.pdf]

Supplementary Table 1 Summary of NHS guidance in England on foods to avoid or limit during pregnancy included in the questionnaire[a]

Food	Guidance	Hazard	Details of hazard	Further information
Meat and meat products				
Game meat and game birds	Avoid	Toxicological	Toxic metal: lead shot and splinters	Lead-shot game meat and gamebirds
Pate (meat and vegetarian)	Avoid	Microbiological, teratogenic	Listeriosis, excess vitamin A	
Cured meats	Avoid	Microbiological	Toxoplasmosis	Avoid cold cured meats such as salami, pepperoni, chorizo and prosciutto unless cooked. Cold pre-packed meats such as ham and corned beef are allowed.
Liver/liver products	Avoid	Teratogenic	Excess vitamin A	
Dairy products				
Soft cheese	Avoid	Microbiological	Listeriosis	Uncooked mould-ripened soft cheeses, uncooked soft blue cheeses, uncooked cheese made from unpasteurised milk
Unpasteurised milk	Avoid	Microbiological	Listeriosis	
Fish				Eat at least two portions per week, one of which should be oily[b]
Shark, marlin, swordfish	Avoid	Toxicological	Toxic metal: mercury	
Oily fish	Limit	Toxicological	Toxic metal: mercury<br>Toxins: dioxins and polychlorinated biphenyls	No more than two portions per week
Tuna	Limit	Toxicological	Toxic metal: mercury	No more than 2 tuna steaks per week or 4 medium-sized cans of tuna (both fresh and tinned tuna do not count as an oily fish)[c]
Supplements				
Multivitamins	Avoid if contain vitamin A	Teratogenic	Excess vitamin A	
Omega-3 supplements	Avoid if derived from fish liver oil	Teratogenic	Excess vitamin A	
Drinks				
Alcohol	Avoid	Toxicological	Adverse birth outcomes, fetal alcohol syndrome	
Caffeinated drinks	Limit	Toxicological		≤200 mg caffeine/day. Caffeine present in coffee, tea, soft drinks, energy drinks, chocolate
Herbal tea	Limit	Pharmacological		No more than 4 cups a day
Miscellaneous				
Hen eggs[c]	(Limit)	Microbiological	Salmonella	Avoid uncooked and partially cooked hens' eggs only if not British-Lion stamped
Peanuts[d]	(Avoid only if nut allergy)	Anaphylactic shock		

For full details of guidance on foods and drinks to limit in pregnancy see NHS website pages[1-10] (guidance on omega-3 supplements[3, 4, 6]).

Updates made to the guidance after the study: (1) previously sushi was acceptable if fish previously frozen, now advised to avoid any raw fish; (2) previously smoked fish (e.g. salmon and trout) was acceptable, now advised that it should be thoroughly cooked. Updated advice accessed 5 September 2022 (updates not included in questionnaire).[3]

[a]Items were not included in questionnaire if they involved guidance on preparation or cooking methods: rare/uncooked meat, unwashed fruits and vegetables, uncooked shellfish, sushi made with fish not previously frozen, rare or uncooked meat, goose/duck/quail eggs. Liquorice root was also not included (oestrogen-like effects).

[b]This guidance is given under a NHS website page on general healthy eating[2], which contains a link to an additional summary of guidance on fish-eating in pregnancy[4].

[c]Previous advice (before 2019): avoid all uncooked or partly cooked eggs (hen, goose, duck and quail).[53]

[d]Previous advice (before 2009): avoid especially if family history of allergy.

Supplementary Table 2 Criteria used for categorisation of adherence (Yes/No) with guidance on foods to avoid or limit in pregnancy

Item	Response category: Adherent	Response category: Not adherent
Game meat/gamebirds<br>Cured meats<br>Soft cheese<br>Unpasteurised milk<br>Alcohol<br>Pate, liver/liver products	Ate or drank before pregnancy but avoided during pregnancy/Don't eat or drink anyway	Ate or Drank more/Ate or Drank same amount/Ate or drank less
Standard multivitamins	Never	Less than once a month/About one to two times per month/About once per week/Several times per week/Once a day
Caffeinated drinks<br>Herbal teas	Drank less/Drank before pregnancy but avoided during pregnancy/Don't drink anyway/Drank same amount	Drank more
Fish	Twice a week/More than twice a week	Never/Less than twice a week
Oily fish	About once a week	Never/Less than once a month/About one to two times a month/Several times a week
Tinned tuna<br>Fresh tuna	Never/Less than once a month/About one to two times a month/About once a week	Several times a week
Shark/marlin/swordfish	Never	Less than once a month/About one to two times per month/About once per week/Several times per week
Hens' eggs<br>Peanuts	Don't eat anyway/Ate same amount/Ate more	Ate less/Ate before pregnancy but avoided during recent pregnancy

Participants responding 'Don't know/Can't remember' were excluded from categorisation.

Supplementary Table 3 Participant characteristics and intakes of foods and drinks with guidance on avoiding consumption during pregnancy (intakes during pregnancy compared with before pregnancy) (maximum n=598)

	All participants						
	**n**	**Don't eat/drink anyway**	**Ate/drank more often**	**Ate/drank same**	**Ate/drank less often**	**Ate/drank before but avoided**	**Chi-square test: p value**
**EDUCATION**[a]							
Soft cheese	594						0.002
Low	114	38 (33%)	1 (1%)	5 (4%)	11 (10%)	59 (52%)	
High	480	91 (19%)	0 (0%)	20 (4%)	44 (9%)	325 (68%)	
Unpasteurised milk	595						
Low	114	98 (86%)	0 (0%)	3 (3%)	2 (2%)	11 (10%)	0.201
High	481	402 (84%)	0 (0%)	4 (1%)	5 (1%)	70 (15%)	
Liver/liver products	590						
Low	113	93 (82%)	0 (0%)	0 (0%)	2 (2%)	18 (10%)	0.007
High	477	302 (63%)	2 (0%)	4 (1%)	8 (2%)	161 (90%)	
Paté (meat/vegetarian)							
Low	124	69 (61%)	0 (0%)	1 (1%)	4 (4%)	40 (35%)	<0.001
High	481	184 (38%)	1 (0%)	8 (2%)	14 (3%)	274 (57%)	
Game meat/gamebirds							
Low	113	89 (78%)	0 (0%)	13 (11%)	5 (1%)	6 (5%)	0.608
High	479	346 (72%)	1 (0%)	69 (14%)	40 (7%)	23 (5%)	
Cured meats							
Low	114	31 (27%)	0 (0%)	26 (23%)	18 (16%)	39 (13%)	<0.001
High	481	88 (18%)	3 (1%)	51 (11%)	77 (16%)	262 (87%)	
Alcohol							
Low	113	30 (26%)	0 (0%)	1 (0%)	9 (8%)	73 (64%)	<0.001
High	482	66 (14%)	0 (0%)	0 (0%)	44 (9%)	372 (77%)	
**AGE (years)**							
Soft cheese	122	38 (31%)	0 (0%)	5 (4%)	11 (9%)	68 (56%)	
<30	472	90 (19%)	1 (0%)	20 (4%)	44 (9%)	317 (67%)	0.067
≥30							
Unpasteurised milk							
<30	122	101 (83%)	5 (4%)	1 (1%)	15 (12%)	15 (12%)	0.009
≥30	473	398 (84%)	2 (0%)	6 (1%)	67 (14%)	67 (14%)	
Liver/liver products							
<30	122	91 (75%)	0 (0%)	2 (2%)	2 (2%)	26 (21%)	0.149
≥30	473	304 (64%)	2 (0%)	2 (0%)	8 (2%)	153 (32%)	
Paté (meat/vegetarian)							
<30	122	68 (56%)	1 (1%)	1 (1%)	1 (1%)	51 (42%)	0.002
≥30	473	185 (39%)	0 (0%)	8 (2%)	18 (4%)	262 (55%)	
Game meat/gamebirds							
<30	122	104 (85%)	0 (0%)	11 (9%)	4 (3%)	2 (2%)	0.021
≥30	473	331 (70%)	1 (0%)	70 (15%)	41 (9%)	28 (6%)	
Cured meats							
<30	122	25 (21%)	2 (2%)	17 (14%)	14 (12%)	64 (53%)	0.181
≥30	473	94 (20%)	1 (0%)	60 (13%)	82 (17%)	236 (50%)	
Alcohol							
<30	123	25 (26%)	0 (0%)	0 (0%)	7 (6%)	90 (73%)	0.105
≥30	473	71 (74%)	0 (0%)	1 (0%)	46 (10%)	355 (75%)	
**HOUSEHOLD INCOME**							
Soft cheese							

	All participants						
	n	Don't eat/drink anyway	Ate/drank more often	Ate/drank same	Ate/drank less often	Ate/drank before but avoided	Chi-square test: p value
<£50,000	263	62 (24%)	0 (0%)	15 (6%)	23 (9%)	163 (62%)	0.135
≥£50,000	296	52 (18%)	1 (0%)	9 (3%)	31 (11%)	203 (69%)	
Unpasteurised milk							0.559
<£50,000	264	218 (46%)	0 (0%)	5 (2%)	3 (1%)	38 (14%)	
≥£50,000	296	252 (53%)	0 (0%)	2 (1%)	4 (1%)	38 (13%)	
Liver/liver products							
<£50,000	264	188 (24%)	2 (1%)	4 (2%)	5 (2%)	62 (24%)	0.001
≥£50,000	296	178 (38%)	0 (0%)	0 (0%)	3 (1%)	113 (38%)	
Paté (meat/vegetarian)							
<£50,000	264	123 (47%)	1 (0%)	7 (3%)	7 (3%)	126 (47%)	0.037
≥£50,000	296	112 (38%)	0 (0%)	2 (1%)	8 (3%)	174 (38%)	
Game meat/gamebirds							
<£50,000	264	212 (80%)	0 (0%)	26 (10%)	16 (6%)	8 (3%)	0.011
≥£50,000	296	198 (67%)	1 (0%)	52 (18%)	26 (9%)	18 (6%)	
Cured meats							
<£50,000	264	59 (22%)	1 (0%)	44 (17%)	38 (14%)	122 (46%)	0.038
≥£50,000	296	49 (17%)	1 (0%)	30 (10%)	51 (17%)	165 (56%)	
Alcohol							
<£50,000	265	51 (19%)	0 (0%)	1 (0%)	18 (7%)	194 (73%)	0.042
≥£50,000	296	36 (12%)	0 (0%)	0 (0%)	33 (11%)	227 (77%)	
**REGIONS**[b]							
Soft cheese							
Northern	153	37 (24%)	0 (0%)	9 (6%)	13 (9%)	94 (61%)	0.835
Midlands	106	25 (24%)	0 (0%)	3 (3%)	11 (10%)	67 (63%)	
Southern	337	67 (20%)	0 (0%)	13 (4%)	31 (9%)	225 (67%)	
Unpasteurised milk							
Northern	153	127 (83%)	0 (0%)	4 (3%)	1 (1%)	21 (14%)	0.617
Midlands	106	89 (84%)	0 (0%)	1 (1%)	1 (1%)	15 (14%)	
Southern	338	285 (84%)	0 (0%)	2 (1%)	5 (2%)	46 (14%)	
Liver/liver products							
Northern	153	99 (65%)	1 (1%)	2 (1%)	3 (2%)	47 (31%)	0.475
Midlands	106	75 (71%)	0 (0%)	2 (2%)	0 (0%)	28 (26%)	
Southern	338	222 (66%)	1 (0%)	0 (0%)	7 (2%)	105 (31%)	
Paté (meat/vegetarian)							
Northern	153	64 (42%)	1 (1%)	3 (2%)	5 (3%)	80 (52%)	0.732
Midlands	106	41 (39%)	0 (0%)	2 (2%)	2 (2%)	61 (58%)	
Southern	338	148 (44%)	0 (0%)	4 (1%)	12 (4%)	174 (52%)	
Game meat/gamebirds							
Northern	153	113 (74%)	0 (0%)	26 (17%)	8 (5%)	5 (3%)	0.307
Midlands	106	85 (80%)	0 (0%)	8 (8%)	6 (6%)	6 (6%)	
Southern	339	238 (70%)	1 (0%)	48 (14%)	31 (9%)	19 (6%)	
Cured meats							0.071
Northern	153	28 (18%)	2 (1%)	24 (16%)	23 (15%)	76 (50%)	
Midlands	106	25 (24%)	0 (0%)	21 (20%)	16 (15%)	44 (42%)	
Southern	338	66 (20%)	1 (0%)	32 (10%)	57 (17%)	182 (54%)	
Alcohol							
Northern	153	21 (14%)	0 (0%)	0 (0%)	10 (7%)	122 (80%)	0.802
Midlands	106	18 (17%)	0 (0%)	0 (0%)	9 (9%)	79 (75%)	
Southern	339	58 (17%)	0 (0%)	1 (0%)	34 (10%)	245 (72%)	
**PARITY**							
Soft cheese							

	All participants						
	n	Don't eat/drink anyway	Ate/drank more often	Ate/drank same	Ate/drank less often	Ate/drank before but avoided	Chi-square test: p value
1	430	89 (69%)	0 (0%)	14 (3%)	38 (9%)	289 (67%)	0.079
≥2	165	40 (31%)	1 (0%)	11 (7%)	17 (10%)	96 (58%)	
Unpasteurised milk							
1	431	364 (85%)	0 (0%)	4 (1%)	4 (1%)	59 (14%)	0.643
≥2	165	136 (82%)	0(0%)	3 (2%)	3 (2%)	23 (14%)	
Liver/liver products							
1	431	291 (68%)	0 (1%)	2 (0%)	6 (1%)	127 (30%)	0.097
≥2	165	105 (64%)	2 (1%)	2 (1%)	4 (2%)	52 (32%)	
Paté (meat/vegetarian)							
1	431	183 (43%)	1 (0%)	4 (1%)	14 (3%)	229 (53%)	0.413
≥2	165	70 (42%)	0 (0%)	5 (3%)	5 (3%)	85 (52%)	
Game meat/gamebirds							
1	428	313 (73%)	1 (0%)	55 (13%)	34 (8%)	25 (5%)	0.448
≥2	165	122 (74%)	0 (0%)	27 (16%)	11 (7%)	5 (3%)	
Cured meats							
1	431	82 (19%)	2 (1%)	43 (10%)	70 (16%)	234 (54%)	0.004
≥2	165	37 (22%)	1 (1%)	34 (21%)	26 (16%)	67 (41%)	
Alcohol							
1	432	67 (16%)	0 (0%)	1 (0%)	36 (8%)	328 (76%)	0.349
≥2	165	30 (18%)	0 (0%)	0 (0%)	17 (10%	117 (71%)	
**SPECIAL DIET**							
Soft cheese							
Yes	121	35 (59%)	0 (0%)	5 (4%)	10 (8%)	71 (59%)	0.288
No	475	94 (66%)	1 (0%)	20 (4%)	45 (10%)	315 (66%)	
Unpasteurised milk							
Yes	121	109 (90%)	0 (0%)	1 (1%)	1 (1%)	10 (8%)	0.230
No	476	372 (82%)	0 (0%)	6 (1%)	6 (1%)	72 (15%)	
Liver/liver products							
Yes	121	19 (16%)	1 (1%)	0 (0%)	4 (3%)	19 (16%)	0.002
No	476	161 (34%)	1 (0%)	4 (1%)	6 (1%)	161 (34%)	
Paté (meat/vegetarian)							
Yes	121	72 (60%)	0 (0%)	3 (3%)	6 (5%)	40 (33%)	<0.001
No	476	181 (38%)	1 (0%)	6 (1%)	13 (3%)	275 (58%)	
Game meat/gamebirds							
Yes	121	104 (86%)	1 (1%)	12 (10%)	3 (3%)	1 (1%)	<0.001
No	476	332 (70%)	0 (0%)	70 (15%)	42 (9%)	29 (6%)	
Cured meats							
Yes	121	65 (32%)	1 (1%)	7 (6%)	9 (7%)	39 (7%)	<0.001
No	476	54 (55%)	2 (0%)	70 (15%)	87 (18%)	263 (18%)	
Alcohol							
Yes	122	23 (19%)	0 (0%)	0 (0%)	12 (10%)	87 (71%)	0.818
No	476	74 (16%)	0 (0%)	1 (0%)	41 (9%)	359 (75%)	

Participants responding 'Don't know/Can't remember' were excluded from analysis.
[a]Low=None/GCSE/Vocational level 1 and 2/AS or A level/Vocational level 3; High=University degree (BSc, BA)/Professional qualification/Vocational levels 4 and 5/University higher degree (MA, MSc, PhD)).
[b]Northern=North East/North West/Yorkshire and Humberside; Midlands=East Midlands/West Midlands; Southern=East/Greater London/South East/South West.

Supplementary Table 4 Associations between demographic characteristics and adherence to guideline for each food/drink item for which guidance is to avoid or limit (odds ratio and 95% confidence interval)

	**Age**	**Education**	**Income**	**Region**	**Parity**	**Special diet**	**Ethnicity**
ALL PARTICIPANTS							
Cured meats	1.13 (0.77, 1.66)	1.60 (0.97, 2.62)	1.06 (0.62, 1.81)	1.16 (0.93, 1.47)	0.68 (0.45, 1.04)	**0.31 (0.17, 0.56)**	0.91 (0.38, 2.19)
Game meat	0.59 (0.32, 1.10)	1.06 (0.45, 2.47)	0.50 (0.16, 1.52)	**0.62 (0.41, 0.93)**	0.79 (0.41, 1.53)	0.53 (0.22, 1.29)	3.45 (0.45, 26.65)
Gamebirds	0.53 (0.24, 1.20)	1.75 (0.63, 4.84)	0.64 (0.17, 2.40)	0.87 (0.52, 1.46)	0.62 (0.26, 1.49)	0.59 (0.17, 2.04)	1.34 (0.17, 10.59)
Soft cheese	1.01 (0.62, 1.65)	1.19 (0.62, 2.27)	1.22 (0.60, 2.50)	1.02 (0.76, 1.37)	0.66 (0.39, 1.11)	0.93 (0.50, 1.73)	1.34 (0.39, 4.60)
Unpasteurised milk	2.07 (0.69, 6.22)	2.54 (0.73, 8.80)	0.82 (0.19, 3.48)	1.38 (0.72, 2.64)	0.46 (0.15, 1.45)	0.78 (0.17, 3.58)	0.60 (0.07, 5.21)
Shark/marlin/swordfish[a]	-	-	-	-	-	-	-
Alcohol	0.75 (0.41, 1.36)	1.17 (0.53, 0.28)	0.87 (0.35, 2.17)	0.69 (0.47, 1.01)	0.91 (0.47, 1.77)	1.14 (0.56, 2.32)	20×10^{7} (0.00, .)
Paté (meat/vegetarian)	**0.37 (0.17, 0.83)**	1.24 (0.42, 3.62)	1.07 (0.33, 3.4)	1.14 (0.70, 1.85)	0.90 (0.37, 2.20)	1.71 (0.69, 4.26)	1.34 (1.17, 10.58)
Liver/liver products	1.02 (0.34, 3.10)	0.78 (0.15, 3.90)	0.85 (0.17, 4.34)	1.26 (0.66, 2.40)	0.37 (0.12, 1.15)	1.78 (0.54, 5.87)	45×10^{6} (0.00, .)
Standard multivitamins	1.81 (0.76, 4.28)	1.05 (0.34, 3.21)	0.78 (0.23, 2.64)	1.25 (0.77, 2.02)	**0.38 (0.16, 0.88)**	1.16 (0.42, 3.22)	0.56 (0.11, 2.80)
Caffeinated drinks							
Soft drinks	1.31 (0.82, 2.10)	**2.25 (1.28, 3.94)**	1.04 (0.75, 2.61)	1.19 (0.90, 1.57)	**0.51 (0.31, 0.84)**	1.30 (0.74, 2.28)	0.75 (0.26, 2.11)
Tea	0.70 (0.38, 1.29)	**3.53 (1.70, 7.40)**	1.38 (0.61, 3.15)	0.96 (0.64, 1.44)	**0.47 (0.24, 0.92)**	0.94 (0.40, 2.24)	**0.27 (0.09, 0.81)**
Coffee	0.58 (0.25, 1.31)	1.96 (0.72, 5.36)	1.19 (0.39, 3.65)	1.05 (0.63, 1.76)	**0.28 (0.11, 0.69)**	0.97 (0.32, 2.98)	0.50 (0.11, 2.35)
Energy drinks	3.79 (0.07, 198.55)	0.00 (0.00, .)	0.00 (0.00, .)	0.63 (0.09, 4.33)	4×10^{6} (0.00, .)	3.42 (0.21, 55.70)	4×10^{19}
Herbal tea	0.86 (0.61, 1.23)	0.91 (0.56, 1.47)	0.61 (0.37, 1.03)	0.76 (0.62, 0.94)	1.07 (0.69, 1.64)	1.07 (0.69, 1.64)	0.49 (0.22, 1.13)
Fish	**1.51 (1.02, 2.25)**	1.35 (0.76, 2.39)	1.01 (0.57, 1.90)	**1.37 (1.07, 1.76)**	1.29 (0.83, 1.98)	0.98 (0.60, 1.59)	0.98 (0.41, 2.32)
Oily fish	**1.64 (1.05, 2.56)**	**2.06 (1.03, 4.12)**	0.66 (0.35, 1.25)	1.16 (0.88, 1.51)	0.71 (0.42, 1.19)	0.80 (0.48, 1.34)	0.44 (0.13, 1.50)
Tinned tuna	1.72 (0.49, 6.01)	1.98 (0.48, 8.18)	0.32 (0.04, 2.79)	1.49 (0.74, 3.02)	1.96 (0.40, 9.54)	0.38 (0.05, 3.03)	0.41 (0.05, 3.50)
Fresh tuna[a]	-	-	-	-	-	-	-
Hens' eggs	**1.50 (0.92, 2.42)**	**1.94 (1.08, 3.47)**	0.64 (0.32, 1.28)	1.12 (0.85, 1.48)	1.82 (1.01, 3.26)	0.83 (0.46, 1.51)	2.09 (0.48, 9.11)
Peanuts	1.71 (0.86, 3.39)	1.16 (0.50, 2.71)	0.88 (0.35, 2.21)	1.04 (0.70, 1.53)	1.39 (0.63, 3.03)	0.53 (0.20, 1.40)	0.93 (0.21, 4.14)
CONSUMERS PRE-PREGNANCY							
Cured meats	1.06 (0.71, 1.58)	**2.18 (1.26, 3.77)**	1.14 (0.64, 2.03)	1.16 (0.91, 1.48)	0.61 (0.40, 0.96)	0.67 (0.34, 1.30)	0.86 (0.34, 2.18)
Game meat	0.88 (0.41, 1.86)	1.02 (0.36, 3.11)	0.83 (0.22, 3.11)	0.63 (0.39, 1.01)	**0.43 (0.19, 0.99)**	0.95 (0.29, 3.09)	1.08 (0.47, 35.53)
Gamebirds	0.92 (0.33, 2.57)	2.67 (0.66, 10.77)	0.89 (0.18, 4.45)	0.90 (0.50, 1.60)	0.46 (0.16, 1.33)	0.74 (0.15, 3.74)	1.59 (0.18, 14.37)
Soft cheese	1.00 (0.61, 1.66)	1.36 (0.70, 2.64)	1.22 (0.60, 2.50)	1.06 (0.78, 1.44)	0.61 (0.35, 1.04)	1.06 (0.56, 2.03)	1.30 (0.37, 4.59)

	Age	Education	Income	Region	Parity	Special diet	Ethnicity
Unpasteurised milk	2.21 (0.60, 8.13)	**5.59 (1.03, 30.25)**	0.57 (0.09, 3.69)	1.67 (0.75, 3.72)	0.45 (0.11, 1.81)	1.42 (0.20, 10.25)	1.26 (0.06, 24.78)
Shark/marlin/swordfish[a]	-	-	-	-	-	-	-
Alcohol	0.77 (0.42, 1.42)	1.36 (0.61, 3.02)	1.03 (0.41, 2.58)	**0.66 (0.45, 0.98)**	0.87 (0.44, 1.70)	1.18 (0.57, 2.42)	228×10^6 (0.00, .)
Paté (meat/vegetarian)	**0.47 (0.21, 1.08)**	1.56 (0.49, 4.93)	1.38 (0.40, 4.77)	1.12 (0.68, 1.87)	0.80 (0.32, 1.98)	**2.70 (1.03, 7.11)**	0.76 (0.09. 6.66)
Liver/liver products	1.47 (0.45, 4.76)	1.24 (0.23, 6.76)	1.30 (0.23, 7.28)	1.31 (0.66, 2.60)	0.42 (0.13, 1.35)	**3.85 (1.03, 14.44)**	76×10^6 (0.00, .)
Caffeinated drinks							
Soft drinks	1.25 (0.77, 2.04)	**2.20 (1.24, 3.93)**	1.30 (0.70, 2.42)	1.16 (0.87, 1.55)	**0.47 (0.28, 0.79)**	1.38 (0.77, 2.48)	0.78 (0.26, 2.31)
Tea	0.65 (0.37, 1.23)	**3.28 (1.53, 7.06)**	1.72 (0.73, 4.08)	0.88 (0.58, 1.33)	**0.45 (0.22, 0.91)**	1.23 (0.50, 3.02)	**0.31 (0.10, 0.95)**
Coffee	0.62 (0.27, 1.43)	1.96 (0.70, 5.51)	1.60 (0.49, 5.21)	1.00 (0.58, 1.72)	**0.30 (0.12, 0.75)**	0.88 (0.28, 2.74)	0.54 (0.11, 2.69)
Energy drinks[a]	-	-	-	-	-	-	-
Herbal tea	0.78 (0.46, 1.33)	1.00 (0.47, 2.13)	0.72 (0.33, 1.59)	0.83 (0.61, 1.14)	0.62 (0.32, 1.19)	0.59 (0.34, 1.05)	0.84 (0.27, 2.61)
Fish	1.47 (0.96, 2.24)	1.64 (0.89, 3.03)	1.06 (0.56, 2.01)	**1.36 (1.05, 1.77)**	1.37 (0.87, 2.15)	0.57 (0.33, 1.01)	1.19 (0.48, 2.95)
Oily fish	1.41 (0.87, 2.30)	**2.50 (1.14, 5.70)**	0.56 (0.27, 1.15)	1.08 (0.81, 1.44)	0.72 (0.42, 1.25)	**0.46 (0.25, 0.84)**	0.42 (0.12, 1.47)
Tinned tuna	3.39 (0.70, 16.37)	0.70 (0.15, 4.98)	0.34 (0.04, 3.14)	1.52 (0.71, 3.26)	1.52 (0.71, 3.26)	0.00 (0.00, 0.00)	0.37 (0.39, 3.39)
Fresh tuna[a]	-	-	-	-	-	--	
Hens' eggs	1.56 (0.96, 2.54)	**1.88 (1.05, 3.38)**	0.69 (0.34, 1.38)	1.09 (0.82, 1.45)	1.78 (0.99, 3.20)	0.88 (0.49, 1.61)	2.10 (0.48, 9.24)
Peanuts	**1.78 (0.90, 3.53)**	1.36 (0.58, 3.20)	0.98 (0.39, 2.50)	1.11 (0.75, 1.63)	1.49 (0.19, 1.29)	0.49 (0.19, 1.29)	0.95 (0.21, 4.34)

Participants responding 'Don't know/Can't remember' were excluded from analysis.

Reference category: Not adherent (see Supplementary Table 2 for further information).

Odds ratios where CI do not cross 1.00 are shown in bold.

Adjusted for: Education (None/GCSE/A levels/Vocational 1-3 (reference), Degree/Higher Degree/Vocational 4-5), Maternal age (18-25 (reference), >25-35, >35 years), Household income (≤£50,000 (reference), >£50,000), Region (Northern (reference), Midlands, Southern), Parity (One (reference), More than one), Special diet (No (reference), Yes), Maternal age (18-25 (reference), >25-35, >35 years) , Ethnicity (White (reference), Other).

[a]Model failed to converge or was very poor fit (low case numbers).
